# Depression risk in chronic tonsillitis patients underwent tonsillectomy: a global federated health network analysis

**DOI:** 10.7150/ijms.93977

**Published:** 2024-03-31

**Authors:** Hui-Chin Chang, Hsin-Yo Lu, Yu-Chen Guo, Chen-Yu Lin, Shiu-Jau Chen, Shuo-Yan Gau

**Affiliations:** 1 Evidence-based Medicine Center, Chung Shan Medical University Hospital, Taichung, Taiwan.; 2 Library, Chung Shan Medical University Hospital, Taichung, Taiwan.; 3 School of Medicine, Chung Shan Medical University, Taichung, Taiwan.; 4 Department of Neurosurgery, Mackay Memorial Hospital, Taipei, Taiwan.; 5 Department of Medicine, Mackay Medical College, New Taipei City, Taiwan.; 6 Department of Medical Education, Kaohsiung Chang Gung Memorial Hospital, Kaohsiung, Taiwan.; 7 Orthopedics Department, Chi-Mei Medical Center, Tainan, Taiwan.

**Keywords:** depression, tonsillectomy, cohort, epidemiology, electronic medical records

## Abstract

**Background:** Tonsillectomy is a common surgery in the US, with possible postoperative complications. While small studies indicate postoperative depressive symptoms may occur, large-scale evidence is lacking on the tonsillectomy-depression link.

**Methods:** We conducted a retrospective cohort study using the TriNetX US collaborative network, offering de-identified electronic health data from 59 collaborative healthcare organizations (HCOs) in the United States. In this study, people being diagnosed of chronic tonsillitis between January 2005 and December 2017 were enrolled. Patients deceased, with previous record of cancers or psychiatric events before index date were excluded. 14,874 chronic tonsillitis patients undergoing tonsillectomy were propensity score matched 1:1 to controls for age, sex, and race. New-onset depression risks were evaluated over 5 years post-tonsillectomy and stratified by age and sex. Confounders were adjusted for including demographics, medications, comorbidities and socioeconomic statuses.

**Results:** After matching, the difference of key baseline characteristics including age, sex, comedications status and obesity status was insignificant between tonsillectomy and non-tonsillectomy groups. Tonsillectomy had a 1.29 times higher 5-year depression risk versus matched controls (95% CI, 1.19-1.40), with elevated risks seen at 1 year (HR=1.51; 95% CI, 1.28-1.79) and 3 years (HR=1.30; 95% CI, 1.18-1.43). By stratifications, risks were increased for both males (HR=1.30; 95% CI, 1.08-1.57) and females (HR=1.30; 95% CI, 1.18-1.42), and significantly higher in ages 18-64 years (HR=1.37; 1.26-1.49), but no significance observed for those 65 years and older. After performing sensitivity analyses and applying washout periods of 6, 12, and 36 months, the outcome remained consistent with unadjusted results.

**Conclusion:** This real-world analysis found tonsillectomy was associated with a 30% higher 5-year depression risk versus matched non-tonsillectomy patients with chronic tonsillitis. Further mechanistic research is needed to clarify the pathophysiologic association between depression and tonsillectomy. Depression is not commonly mentioned in the current post-tonsillectomy care realm; however, the outcome of our study emphasized the possibility of these suffering condition after operation. Attention to psychological impacts following tonsillectomy is warranted to support patient well-being, leading to better management of post-tonsillectomy individuals.

## Introduction

Tonsillectomy is one of the most common surgeries in the United States, with 399,000 procedures performed in patients under the age of 44 annually based on the published data 1. The surgical procedure involves full or partial removal of the palatine tonsils, and is often carried out with adenoids (nasopharyngeal) removal simultaneously 2. Indications for surgery comprise obstructive sleep-disordered breathing and recurrent throat infections, both of which can bring formidable impact on the quality of life and health status in people of all ages 3. Some studies have shown that tonsillectomy can have a positive effect on a patient's life pattern including limitation in sleep, usual activities, vitality and discomfort, resulting in statistically improving their life quality 4-6. However, complications such as postoperative nausea and vomiting, throat pain, dehydration, and bleeding are included after the surgical procedure 7, 8.

Depression or major depression disorder is a heterogeneous disease characterized by loss of energy, loss of pleasure and interest in usually enjoyable activities, low mood, difficulties in solving problems and thinking, suicidal intention and psychomotor disturbances 9. This mental disease has an estimated average lifetime prevalence of 15% in well-developed countries 10. The pathogenesis of MDD still remains unknown, however, from the prospection of neurobiology, the degradation, inflammation, or changes in brain structures such as nucleus accumbens, hypothalamus, and frontal regions may be associated with the occurrence of this disease 11. Form another point of view, negative life events, absence of parents, low educational attainment, low socioeconomic status, and many other unmentioned environmental conditions could be risk factors of MDD as well 12. Furthermore, the frequent recurrence and seriousness of the disorder may not only cause reduction to the life quality and normal functioning, but may also lead to higher mortality and physical morbidity 13. One study indicates that tonsillectomy can possibly help improve depression in children with obstructive sleep apnea syndrome after treatment 14. However, we hypothesize that the postoperative pain and stress from tonsillectomy can lead to depression. Though the pathogenesis and connection between tonsillectomy and depression are not yet illuminated, there are some possible conditions that may interweave these diseases. The postoperative pain caused by tonsillectomy sometimes could be severe and intolerable, and one research indicates that acute pain can alter synaptic connectivity and dopamine signaling in brain, thus turn out to be a risk factor of depression. On the other hand, depression could lower the threshold of pain sensation, leading to a vicious cycle 15, 16. If the importance of controlling postoperative pain is ignored by patients, it could become chronic pain and eventually damage tissue, nerve, and may even cause complex region pain syndrome 17. Therefore, enhancing postoperative monitoring, medical care, and other pain-control support should be taken into consideration to improve patient outcomes.

Though case reports and small-scale studies indicated that post-tonsillectomy depressive symptoms could occur after operation 18, 19, large-scale real-world evidences in were lacking. Therefore, we conducted a multicenter, retrospective cohort study to discusses depression risk after tonsillectomy over 5 years, supported by statistics based on large dataset. We used propensity score matching to help us compare the possibility of having depression between patients underwent tonsillectomy and controls after matching covariance. Moreover, we performed sensitivity analysis by applying models including changing variables to improve credibility. The primary objective of this study is to evaluate the risk of depression in patients underwent tonsillectomy over 5 years follow-up time using a large electronic medical record dataset.

## Methods

### Data source

The research utilized a retrospective cohort design and was conducted across several healthcare centers. The source of the analyzed data were obtained through the TriNetX research network, offering de-identified electronic health records from collaborative healthcare organizations (HCOs) globally. This extensive federated database comprised over 120 collaborative HCOs and has been employed for conducting epidemiological studies across diverse fields 20, 21. In TriNetX, the HCOs represents different regions and countries, including the United States, UK, Spain, India, Taiwan, etc. Available de-identified health information including claims, biomarkers, lab data, tumor registries and electronic health records were provided by HCOs. These HCOs periodically update their information, typically on a weekly, bi-weekly, or monthly basis. We utilized the subset “US collaborative network” in this study and retrieved electronic health records only from HCOs in the United States. In the US collaborative network, there were 59 HCOs and electronic health records of more than 100 million patients were available. For disease identification, International Classification of Diseases, Tenth Revision, Clinical Modification, (ICD-10-CM codes) were applied. In the current study, ICD codes were utilized to identify statuses including chronic tonsillitis, neoplasms, depression, schizophrenia, suicide attempt, bipolar disorder, diabetes mellitus, hypertension, hyperlipidemia, problem in socioeconomic and psychosocial circumstances and substance abuse. Anatomical Therapeutic Chemical (ATC) codes were applied for identification of medication use. Comedications including antidepressants and corticosteroids were identified based on the record of ATC codes. Moreover, Current Procedural Terminology (CPT) codes were applied for procedure history. Records of tonsillectomy was identified based on CPT codes. Descriptions of the specific codes used in our analysis can be found in **Table S1.**

### Study population and outcome measurement

Enrollment period was set between January 2005 and December 2017. Within this period, people being diagnosed of chronic tonsillitis were enrolled in this study. Given that TriNetX was a prospectively updated international database, all of the enrolled participants could possess a minimum follow-up period of five years. In the main analysis, the follow-up duration starts from 30 days after index date and ends at the follow-up period of 1, 3 and 5 years after the start point. People with chronic tonsillitis and had record of underwent tonsillectomy were enrolled as tonsillectomy group. In the current study, patients with chronic tonsillitis were defined as having the diagnosis record of ICD-10-CM, J35.01, J35.02, J35.03 or J36. Tonsillectomy was defined as having the record of CPT code of 1007181. People with chronic tonsillitis and had no experience of tonsillectomy before or on the index date were recruited as control group. To avoid potential frailty bias and confounding bias, in both groups, all patient deceased before index date and people with previous record of cancers or psychiatric events (including depression, schizophrenia, bipolar disorder or suicide attempts) before index date would be excluded. To further address confounding bias, we utilized propensity score matching in our analyses. Covariates of propensity score matching includes confounders such as age (continuous), sex (male or female), race (White, Black, Asian…etc), status of obesity (greater or lesser than 35) , diabetes mellitus (yes or no), hypertension(yes or no), hyperlipidemia(yes or no), antidepressants use (yes or no), corticosteroids use (yes or no), status of smoking, alcoholism and substance use (yes or no), medical utilization (history of inpatient visit; history of ambulatory visit) and CRP level (greater or less than 3 mg/L) were covariates for matching in the current study. The endpoint of the follow up was set as new-onset depression. The outcome event will be evaluated base on follow-up time of 1, 3 and 5 years after index date. After matching, there were 14,874 people enrolled in the tonsillectomy group and the same amount of controls enrolled.

### Stratification and Sensitivity analyses

To evaluate whether age and sex difference influences the association between tonsillectomy and new-onset depression, stratification analysis based on age and sex subgroups has been performed. Age stratification was stratified based on two age subgroups: people between 18 and 64 years old, people greater than 65 years old. Sex stratification was developed based on two sex subgroups: male and female. Sensitivity analyses has been performed based on different model of follow-up time and matching covariates to validate the observed association.

### Statistical analysis

All statistical analysis was performed in the function of Analytics in the TriNetX research network. While comparing the baseline characteristics, standardized difference (SD) was applied to present the difference between groups. SD value greater than 0.1 represents a significant difference. The calculation of the propensity scores was performed using the previously mentioned covariates. Hazard ratio (HR) was calculated to determine the risk of future depression in each group, utilizing Cox proportional hazard model. To determine the significance, 95% confidence intervals (95% CI) were simultaneously calculated while evaluating HR in each analysis.

### Statement of ethics

All analyses performed in the current study did not involve any intervention or interaction with human participants. Dataset in TriNetX research network was de-identified abiding the standard mentioned in the *Section §164.514(a) of the HIPAA Privacy Rule* and has been attested by a qualified expert as defined in* Section §164.514(b)(1) of the HIPAA Privacy Rule.* This official decision, made by an accredited specialist and updated in December 2020, replaces the necessity for TriNetX's prior exemption granted by the Western Institutional Review Board (IRB)^17^, ^22^. Hence, studies utilizing TriNetX research network were exempted from IRB permission. This study abided by the STROBE guideline and the TriNetX publication guideline 17.

## Results

**Figure 1:**
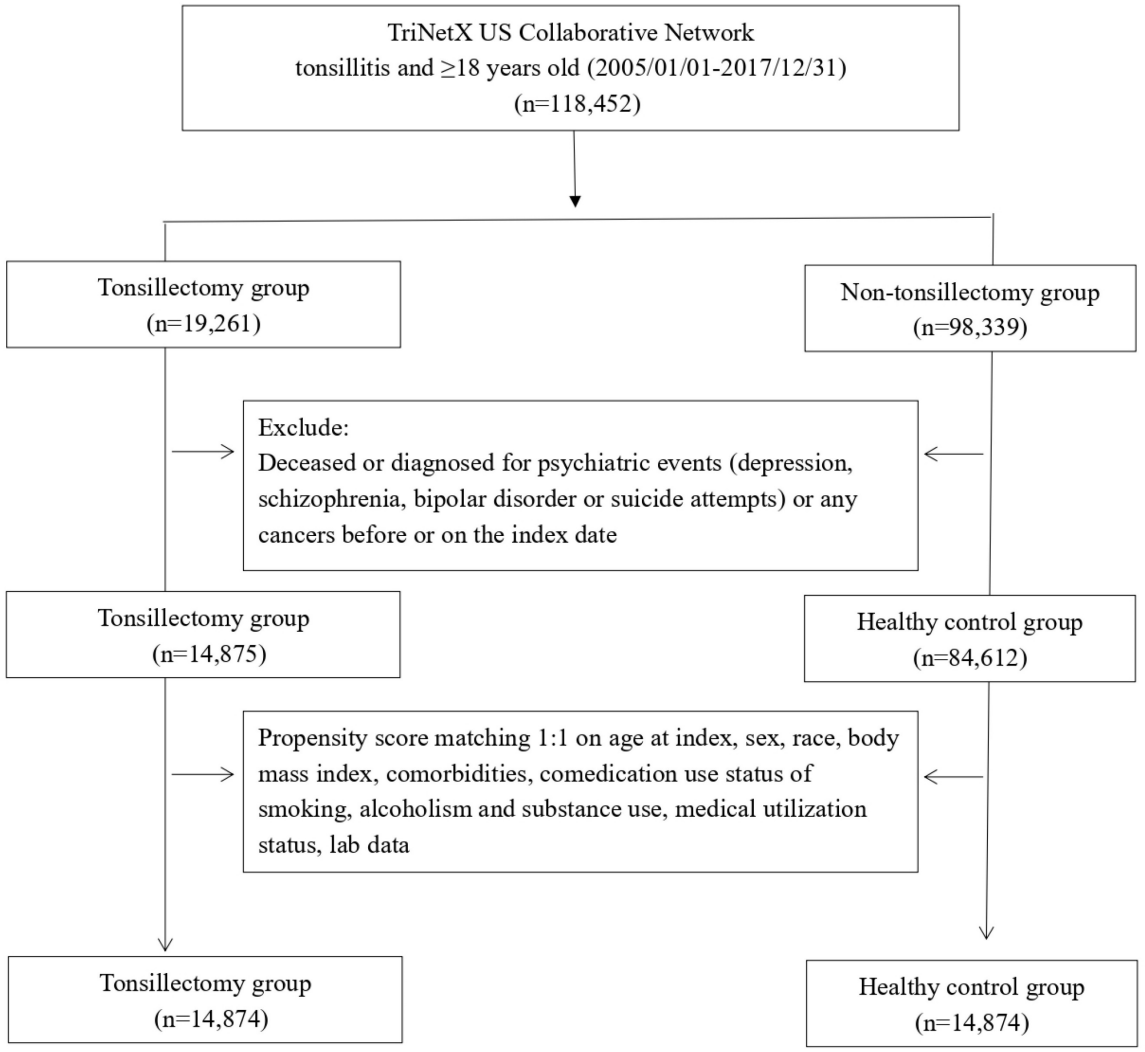
Flowchart of participant selection.

After propensity score matching, there were 14,874 patients enrolled in the tonsillectomy group and the same number of patients enrolled in non-tonsillectomy group (**Figure 1**). The average age of participants in tonsillectomy group was 24 years old and 67.3% of them were female. Before matching, some of the critical baseline characteristics including age, sex, comedications status and obesity status was different between the two groups. In the tonsillectomy cohort, male consisted of 32.2% of population, whereas in the control group, 43.3% of the population was male. As for race distribution, 62.6% of the population in the tonsillectomy cohort was white before matching. In the control group, the proportion of white people was 60.5%. 6.4% of people in the tonsillectomy cohort once prescribed of antidepressants. However, in the control group, the ratio was 3.0%. The difference become insignificant after performing matching.

People underwent tonsillectomy were associated with high risk in having depression within five years after the surgery, with a 1.29-fold risk comparing with people in the non-tonsillectomy group (95% CI, 1.19-1.40). The hazard ratios were 1.51 (95% CI 1.28-1.79) at 1 year, 1.30 (95% CI 1.18-1.43) at 3 years follow-up (**Table 2**). Stratified analyses indicated the increased depression risk was consistent for both male (HR=1.30; 95% CI, 1.08-1.57) and female (HR=1.30; 95% CI, 1.18-1.42) tonsillectomy patients relative to their control counterparts (**Table 3**). When stratified by age, the risk was significantly higher in working-age adults ages 18-64 years (HR=1.37; 95% CI, 1.26-1.49), but not those 65 years and older (HR=0.96; 95% CI, 0.51-1.82).

Sensitivity analyses were conducted to evaluate the robustness and internal validity of the tonsillectomy-depression association (**Tables S2-S4**). Considering the potential effect of extraneous variable, various matching variables were applied in sensitivity analyses. Using an unadjusted model and three other propensity score models adjusting for additional sets of confounders, the hazard ratio ranged from 1.28 to 1.78 but remained statistically significant in all models after 5 years follow-up (**Table S2**). Additionally, applying washout periods of 6, 12, and 36 months at the start of follow-up to exclude prevalent cases yielded consistent results, with 5-year hazard ratios around 1.29 (**Table S3**). We have additionally performed an additional sensitivity analysis including only people with obstructive sleep apnea. The observed association remained in this analysis (**Table S4**).

## Discussion

In this study, we investigated the risk of depression within five years after tonsillectomy in patients with chronic tonsillitis. We found that the short-term risk increased by approximately 30%. A further stratified analysis revealed significant differences in the age groups of men, women, and 18 to 64 years of age. These results remained significant even after a thorough consideration of confounding factors such as age, sex, race, and socioeconomic status.

Tonsillectomy is one of the most common surgical procedures around the world, performed in both children [23]and adults 24. It is indicated primarily for chronic tonsil enlargement, chronic tonsillitis, or obstructive sleep apnea. In the United States, tonsillectomy in children ranges from 3.4 to 4.8 cases per 1000 individuals 25, and adults over 15 years of age represent approximately 40% of total tonsillectomy cases 26. Despite its crucial role in improving respiratory function and quality of life, postoperative complications cannot be overlooked. These complications include infection, postoperative pain, bleeding, tonsillar stones, and obstructive sleep apnea (OSA) 24, 27, 28. Over time, various tonsillectomy options have emerged with a total postoperative bleeding rate of 4.60% (95% CI: 2.1-8.0) and an average postoperative pain rating of 3.53 (95% CI: 2.87-4.19) 29.

Previous studies have identified associations between surgeries such as coronary artery bypass grafting, stoma surgery, and oophorectomy, and the risk of postoperative depression 30-32. Patients who underwent coronary artery bypass grafting had a higher risk of developing new-onset depression compared to controls of the same gender and age (aHR: 1.05, 95% CI: 1.01-1.09). The risk of depression was particularly evident in younger patients within the first five years after surgery 33. Unilateral and bilateral oophorectomy increased the overall risk of depression (aHR: 1.36, 95% CI: 1.19-1.55), with significantly higher rates of depression among women under 50 years of age 32. Our study found an increased risk of depression within 5 years after tonsillectomy (aHR: 1.29, 95% CI: 1.19-1.40), which demonstrated a significant increase in risk similar to the aforementioned surgeries, especially in young postoperative patients.

Although the correlation between tonsillectomy and depression has been established in children 19, there is limited exploration in the adult population. To our knowledge, our study represents the first comprehensive analysis of the long-term risk of depression after adult tonsillectomy at the population level. We recommend further study validate the incidence proportions in other databases or study population to evaluate the generalizability of the association between tonsillectomy and depression.

The pathophysiology of depression is complex, involving both sociopsychological and biological components 16. There are numerous plausible theories for the link between tonsillectomy and depression. The surgical procedure may induce pain and concerns about mortality, which affect the patient's mental state. Furthermore, the period of separation from loved ones and work can lead to challenges in adjusting, intensifying feelings of anxiety and depression during the perioperative phase 34.

In addition to the above, the derangements of metabolic and physiological processes that may result from surgery, as well as the post-surgical recovery process, can be considered a source of chronic stress 35. During prolonged exposure to these chronic stress sources, the balance of the hypothalamic-pituitary-adrenocortical (HPA axis) is disrupted, resulting in high levels of corticotrophin-releasing hormone (CRH) and glucocorticoids (GC) 36, 37. The primary endogenous GC that humans have is CORT, whereas in rodents, it is corticosterone 36. In addition, postoperative pain can also be one of the mechanisms. Pain and depression are known to be common complications, and some antidepressants are effective against pain, but the mechanisms between them are still unclear 38. Chronic pain is also considered a type of chronic stress that can also lead to HPA-axis dysregulation 36, 39.

Long-term exposure to high concentrations of GC reduces the number of signals of the hippocampal circuits and the prefrontal cortex (PFC) glucocorticoids through at least two receptors, including the mineralocorticoid receptor (MR) and the glucocorticoid receiver (GR), thereby reducing the negative feedback regulation mechanism of the HPA axis 36, 37, 39. In addition, loss of anti-inflammatory signaling mechanisms and an increase in pro-inflammatory cytokines are produced, resulting in proliferation of peripheral inflammatory events and pain sensitization 40. The HPA axis can also be activated by Cytokines through direct action on the brain 36.

High concentrations of GC can also affect many neurotransmitter systems. MDD and PTSD are characterized by a noradrenergic system that is associated with stress-related affective disorders like MDD and PTSD 37, 41. It has been documented that GCs can reverse the effects of β-adrenergic signaling 41. The 5-HT1A receptor is crucial for the pathogenesis and treatment of depression associated with the HPA axis, as supported by a large amount of evidence 42. In the hippocampus, the activity of the 5-HT1A receptor is largely controlled by MR/GR mediated influence during chronic stress-induced sustained increases of GCs 43. Furthermore, HPA-axis dysregulation over time results in structural modifications, including hypertrophy and adrenal hyperplasia 36, 44.

The relationship between depression and the HPA axis has been widely discussed, with more than 40-60% of depression patients having hypercortisolism or HPA-axis dysregulation 45. Neurotransmitter systems affected by HPA-axis dysregulation, as described above, may alter the patient's physical and mental condition and lead to emotional disorders such as depression, such as the transmission of serotonin (5-HT) and norepinephrine (NE) 41. Additionally, studies have proven that individuals with Cushing's syndrome-induced elevated GC may exhibit emotional symptoms 46. CRH acts primarily through the CRH (1) receptor, resulting in numerous anxiety and depressive symptoms 47. Similarly, the mechanisms of PTSD patients are related to the HPA-axis, except that they exhibit oversensitivity to the HPA axis 37, 48, 49. Although GC levels in PTSD patients are usually relatively low, the two diseases seem to have a common pathological mechanism, namely excessive secretion of central CRH, both of which have increased CRH levels in cerebrospinal fluid 47, 48.

In general, amygdala removal after surgery can cause a variety of psychological and physiological stresses, including postoperative pain, leading to an imbalance of the HPA axis, rising levels of CRH and GC, and disorders in the signal transmission of serotonin (5-HT) and norepinephrine (NE), creating a physical, emotional and psychological burden that may eventually accumulate into symptoms of mental illnesses such as depression and anxiety.

The main benefit of this study is its comprehensive coverage of data from 19 regions around the world and more than 120 health facilities. The results remained stable after conducting sensitivity analyzes using different covariate-matching models. Furthermore, we made an effort to minimize any potential confounding variables to improve the precision of the evidence during the stratified analysis after the match.

It is important to approach these study findings with caution due to a number of limitations. First, in retrospective real-world studies derived from databases, causality between the intervention and the outcome events could not be inferred 50, 51. Second, the use of diagnostic codes and medical records may not entirely capture the true depressive symptoms of patients and identify the mechanisms, suggesting potential incompleteness of the records. Third, due to the limitation of the database, unmeasured confounders could potentially cause biases to the results. Furthermore, there were limited data on patient adherence, family history, use of anesthesia, postoperative recovery status, and socioeconomic and lifestyle factors available for consideration and these unaccounted variables may have had an impact on the observed associations.

Although we considered postoperative pain severity and surgical factors (such as glandular status) as potential confounders in the analysis, we were unable to access this relevant information from the TriNetX database. However, our large sample size may offset overall differences. Future studies can continue to explore whether the severity of pain indirectly affects patients' mental health through other mechanisms and how different surgical factors affect patient recovery and psychological status.

Previous research has shown that there is a connection between feelings of sadness and the severity of worry and pain (which includes limitations in daily activities and pain related to discomfort), as well as pain in muscles and bones, heart, lung, and stomach-related discomfort 52. Additional sources also suggest that a decrease in feelings of sadness and worry after surgery is associated with a reduction in pain after the procedure 53, 54. Furthermore, a recent analysis that combined studies revealed a correlation between catastrophic thinking about pain, fear of experiencing pain, and constant attention to pain, with unpleasant emotions, nervousness, and feelings of sadness related to discomfort 55.

Various surgical factors have been shown to influence both recovery and psychological status. Previous research has shown that there are differences between patients in terms of bleeding rates, pain, and postoperative recovery quality between different types of tonsillectomy 29, 56. Furthermore, a significant amount of literature validates the idea that improved recovery after surgery (ERAS) protocols and similar approaches can reduce postoperative pain, anxiety, and depression, thus improving quality of life 57-59.

Two other avenues for future research can be explored: prospective studies of biomarkers to better understand the detailed biological mechanisms between them and deeper investigations of psychological states following tonsillectomy. This includes psychiatric interviews and evaluations of patient pain perception, quality of life, and social support.

Our understanding of the connection between surgery and depression can be expanded by providing clinical recommendations through this study. It is essential to closely consult and monitor high-risk groups, especially young surgical patients (<65 years), to detect and manage possible mental health problems early, such as postoperative pain management or relaxation techniques. Sharing decision-making after surgery is of great importance, as it can guarantee that patients receive relevant information and support before and after surgery, resulting in more personalized and compassionate medical care. This study not only provides valuable information for future clinical practice but also has significant implications for improving clinical practice and patient care.

## Supplementary Material

Supplementary tables.

## Figures and Tables

**Figure 2:**
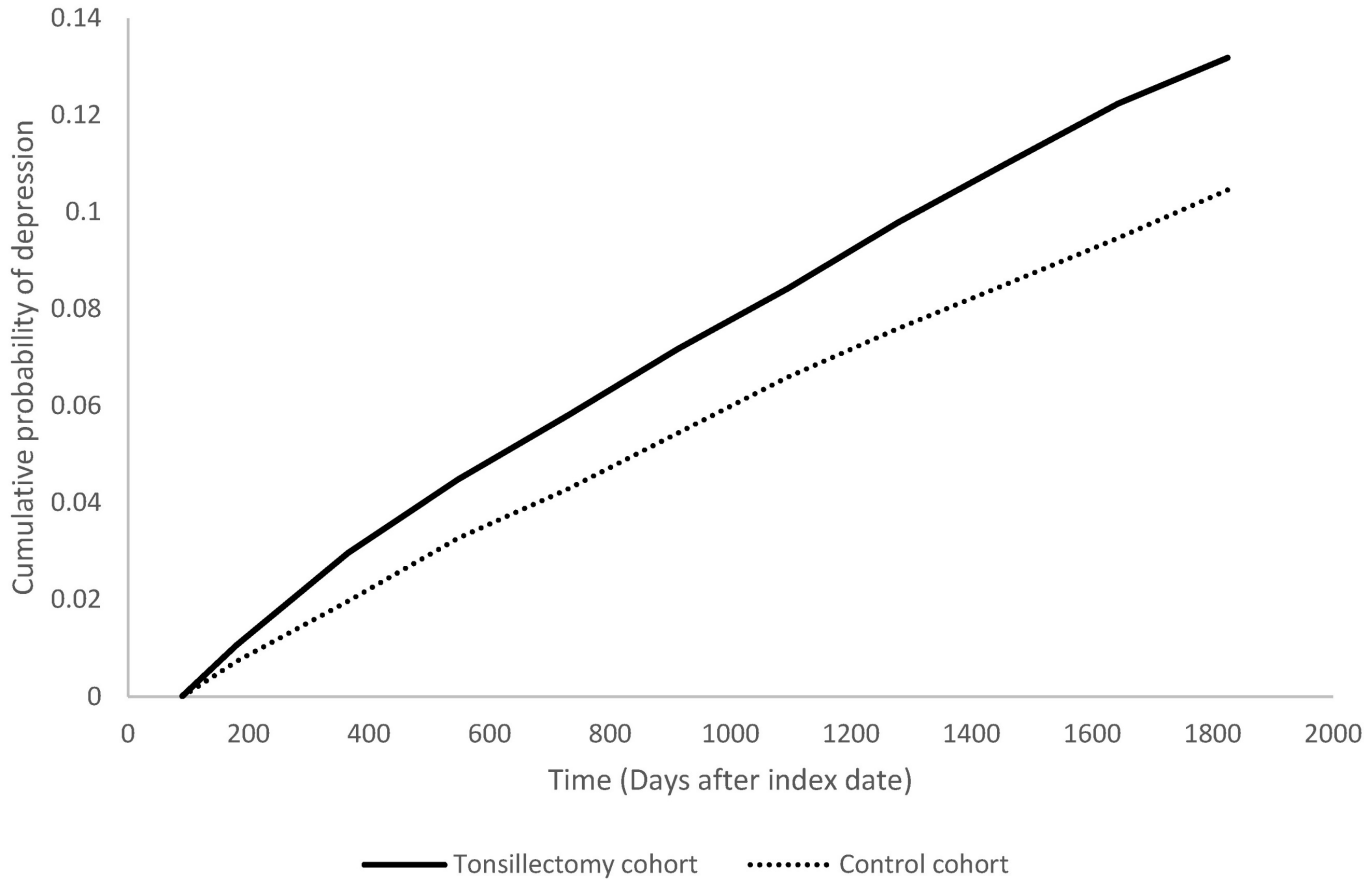
Kaplan Meier plot of depression risk in tonsillectomy cohort and control cohort.

**Table 1:** Baseline characteristics of study subjects (before and after propensity score matching).

	Before matching			After matching^a^		
	Tonsillectomy cohort (n = 14,875)	Control cohort (n = 84,612)	Standardized difference	Tonsillectomy cohort (n = 14,874)	Control cohort (n = 14,874)	Standardized difference
**Age at index**						
Mean ± SD	24.0±10.8	22.1±14.5	**0.15**	24.0±10.8	24.6±12.2	0.05
**Sex**						
Male	4792(32.2)	36597(43.3)	**0.23**	4792(32.2)	4779(32.1)	0.00
Female	10011(67.3)	47858(56.6)	**0.22**	10010(67.3)	10025(67.4)	0.00
**Race, n (%)**						
White	9317(62.6)	51231(60.5)	0.04	9317(62.6)	9350(62.9)	0.00
Black or African American	1801(12.1)	11438(13.5)	0.04	1801(12.1)	1771(11.9)	0.01
Asian	335(2.3)	2314(2.7)	0.03	335(2.3)	331(2.2)	0.00
American Indian or Alaska Native	55(0.4)	360(0.4)	0.01	55(0.4)	50(0.3)	0.01
**Lifestyle**						
Alcohol dependence, smoking and substance use	461(3.1)	2263(2.7)	0.03	461(3.1)	413(2.8)	0.02
**Comorbidities**						
Hypertension	481(3.2)	2463(2.9)	0.02	481(3.2)	391(2.6)	0.04
Diabetes mellitus	214(1.4)	1153(1.4)	0.01	214(1.4)	150(1.0)	0.04
Hyperlipidemia	256(1.7)	1366(1.6)	0.01	256(1.7)	188(1.3)	0.04
**Medications**						
Antidepressants	950(6.4)	2501(3.0)	**0.16**	949(6.4)	877(5.9)	0.02
Corticosteroids	2111(14.2)	6941(8.2)	**0.19**	2110(14.2)	2002(13.5)	0.02
**Medical Utilization Status**						
Ambulatory visit	8499(57.1)	38145(45.1)	**0.24**	8498(57.1)	8451(56.8)	0.01
Inpatient visit	2140(14.4)	10778(12.7)	0.05	2139(14.4)	2061(13.9)	0.02
**Laboratory data**						
BMI, n (%)						
≥ 35 (kg/m^2^)	552(3.7)	1153(1.4)	**0.15**	551(3.7)	455(3.1)	0.04
C reactive protein, n (%)						
≥ 3 (mg/L)	352(2.4)	1245(1.5)	0.07	352(2.4)	315(2.1)	0.02

^a^ Bold font represents a standardized difference was more than 0.1; propensity score matching was performed on age at index, sex, race, body mass index, status of comorbidities (including diabetes mellitus, hypertension, hyperlipidemia), status of comedication use (antidepressants, corticosteroids), status of smoking, alcoholism and substance use, medical utilization status(inpatient, ambulatory), lab data (CRP)

**Table 2:** Risk of depression under different follow-up time^a^

Outcomes	Hazard ratio (95% Confidence interval)^b^		
	1 year	3 years	5 years
Depression	**1.51 (1.28,1.79)**	**1.30 (1.18,1.43)**	**1.29 (1.19,1.40)**

^a^Data present here were the value of follow up from 90 days after index date to the respective following up years. ^b^ Propensity score matching was performed on age at index, sex, race, body mass index, status of comorbidities (including diabetes mellitus, hypertension, hyperlipidemia), status of comedication use (antidepressants, corticosteroids), status of smoking, alcoholism and substance use, medical utilization status(inpatient, ambulatory), lab data (CRP)

**Table 3:** Stratification analysis of depression risk in tonsillectomy patients

	Cases occurring new-onset depression		
Subgroups	Tonsillectomy cohort (No. of event/ Tonsillectomy patient amount in each subgroup)	Control cohort (No. of event/ non-Tonsillectomy patient amount in each subgroup)	HR (95% CI)^a^
**Gender**			
Male	244/4792	194/4792	**1.30 (1.08,1.57)**
Female	1059/10,008	873/10,008	**1.30 (1.18,1.42)**
**Age at index date**			
18-64 years old	1296/14,645	994/14,645	**1.37 (1.26,1.49)**
≥ 65 years old	19/226	19/226	0.96 (0.51,1.82)

^a^ Propensity score matching was performed on age at index, sex, race, body mass index, status of comorbidities (including diabetes mellitus, hypertension, hyperlipidemia), status of comedication use (antidepressants, corticosteroids), status of smoking, alcoholism and substance use, medical utilization status (inpatient, ambulatory), lab data (CRP)
