# Supplementary material for: Depression risk in chronic tonsillitis patients underwent tonsillectomy: a global federated health network analysis
Source: Int J Med Sci. 2024 Mar 31;21(5):949–57. doi: 10.7150/ijms.93977 (PMC11008477; doi:10.7150/ijms.93977)
Supplement: Supplementary file 1 — Supplementary tables. [file ijmsv21p0949s1.pdf]

Supplementary Files

Supplementary Tables

Table S1. Utilized administrative codes in the current study

**Description**	**ICD-10-CM codes [a]**
Chronic tonsillitis	J35.01, J35.02, J35.03, J36
Neoplasms	C00-D49
Depression	F32-F33
Schizophrenia	F31
Suicide attempt	T14.91
Bipolar disorder	F20
Diabetes mellitus	E08-E13
Hypertension	I10
Hyperlipidemia	E78.5
Socioeconomic and psychosocial circumstances	Z55-Z65
Substance abuse	F10-F19
**Medications**	**ATC codes**
Antidepressants	N06A
Corticosteroids	C05AA
**Procedures**	CPT codes
Tonsillectomy	1007181

[a]ICD-10-CM: International Classification of Diseases, Tenth Revision, Clinical Modification

Table S2. Sensitivity analysis: risk of depression in tonsillectomy patients based on different covariate matching models, with 5-year follow up

Outcomes	Hazard ratio (95% Confidence interval)			
	Crude	Model 1[a]	Model 2[c]	Model 3[d]
Depression	**1.78 (1.67,1.89)**	**1.35 (1.24,1.46)**	**1.37 (1.27,1.49)**	**1.28 (1.18,1.38)**

[a] Propensity score matching was performed on age at index, sex

[b] Propensity score matching was performed on age at index, sex, race and comorbidities

[d] Propensity score matching was performed on age at index, sex, race, body mass index, comorbidities, comedication use status of smoking, alcoholism and substance use, medical utilization status, lab data and socioeconomic status

Table S3. Sensitivity analysis: risk of depression with different wash-out periods [a]

Outcomes	Hazard ratio (95% Confidence interval)[b]		
	6 months	12 months	36 months
Depression	**1.30 (1.20,1.41)**	**1.28 (1.18,1.40)**	**1.29 (1.16,1.43)**

[a] Incident events occurred within each wash-out period were excluded in the corresponding analysis, following up for 5 years after index date

[b] Propensity score matching was performed on age at index, sex, race, body mass index, status of comorbidities (including diabetes mellitus, hypertension, hyperlipidemia), status of comedication use (antidepressants, corticosteroids), status of smoking, alcoholism and substance use, medical utilization status (inpatient, ambulatory), lab data (CRP)

Table S4. Sensitivity analysis: risk of depression with different follow-up periods in people with obstructive sleep apnea [a]

Outcomes	Hazard ratio (95% Confidence interval)[b]		
	1-year	3-years	5-years
Depression	**1.92 (1.48,2.48)**	**1.49 (1.27,1.74)**	**1.57 (1.37,1.81)**

[a] Incident events occurred within each wash-out period were excluded in the corresponding analysis, following up for 5 years after index date

[b] Propensity score matching was performed on age at index, sex, race, body mass index, status of comorbidities (including diabetes mellitus, hypertension, hyperlipidemia), status of comedication use (antidepressants, corticosteroids), status of smoking, alcoholism and substance use, medical utilization status (inpatient, ambulatory), lab data (CRP)
